# Temporal Assessment of Self-Regulated Learning by Mining Students’ Think-Aloud Protocols

**DOI:** 10.3389/fpsyg.2021.749749

**Published:** 2021-11-04

**Authors:** Lyn Lim, Maria Bannert, Joep van der Graaf, Inge Molenaar, Yizhou Fan, Jonathan Kilgour, Johanna Moore, Dragan Gašević

**Affiliations:** ^1^TUM School of Education, Technical University of Munich, Munich, Germany; ^2^Behavioural Science Institute, Radboud University, Nijmegen, Netherlands; ^3^School of Informatics, University of Edinburgh, Edinburgh, United Kingdom; ^4^Centre for Learning Analytics, Faculty of Information Technology, Monash University, Melbourne, VIC, Australia

**Keywords:** self-regulated learning, temporal patterns in SRL, process mining, fuzzy miner, think aloud

## Abstract

It has been widely theorized and empirically proven that self-regulated learning (SRL) is related to more desired learning outcomes, e.g., higher performance in transfer tests. Research has shifted to understanding the role of SRL during learning, such as the strategies and learning activities, learners employ and engage in the different SRL phases, which contribute to learning achievement. From a methodological perspective, measuring SRL using think-aloud data has been shown to be more insightful than self-report surveys as it helps better in determining the link between SRL activities and learning achievements. Educational process mining on the basis of think-aloud data enables a deeper understanding and more fine-grained analyses of SRL processes. Although students’ SRL is highly contextualized, there are consistent findings of the link between SRL activities and learning outcomes pointing to some consistency of the processes that support learning. However, past studies have utilized differing approaches which make generalization of findings between studies investigating the unfolding of SRL processes during learning a challenge. In the present study with 29 university students, we measured SRL *via* concurrent think-aloud protocols in a pre-post design using a similar approach from a previous study in an online learning environment during a 45-min learning session, where students learned about three topics and wrote an essay. Results revealed significant learning gain and replication of links between SRL activities and transfer performance, similar to past research. Additionally, temporal structures of successful and less successful students indicated meaningful differences associated with both theoretical assumptions and past research findings. In conclusion, extending prior research by exploring SRL patterns in an online learning setting provides insights to the replicability of previous findings from online learning settings and new findings show that it is important not only to focus on the repertoire of SRL strategies but also on how and when they are used.

## Introduction

A key competence for lifelong learning is self-regulated learning (SRL), otherwise known as “learning to learn,” and it refers to the ability to monitor and adapt one’s learning (European Union, 2019). During SRL, students actively make decisions on the metacognitive and cognitive strategies they deploy to monitor and control their learning to achieve their goals. Yet, students experience difficulties regulating their learning in digital or online settings whereby further support is necessary (Azevedo and Feyzi-Behnagh, 2011; Zheng, 2016; Wong et al., 2019; Poitras et al., 2021). Digital and online learning settings are distinct to traditional classroom learning in that learning tasks, tools, and support are often embedded (e.g., Azevedo et al., 2010; Molenaar et al., 2011; Kinnebrew et al., 2014; Poitras et al., 2021) and students navigate the learning environment autonomously and make decisions as to how their learning takes place and how the learning tasks are completed. Therefore, there needs to be more focus on SRL in digital and online learning, owing to the sharp increase in learning taking place in these settings, largely driven by the ongoing pandemic (EDUCAUSE, 2021). It has been widely theorized and empirically supported that SRL is related to more desired learning outcomes (e.g., performance in transfer tests; Panadero, 2017; Schunk and Greene, 2017). However, SRL consists of complex and dynamic activities and processes which are adapted as students regulate their learning and, therefore, need further investigation in order to support students’ learning (Azevedo et al., 2010; Winne, 2010). Hence, beyond learning outcomes, research has shifted to understanding the role of SRL during learning, such as the strategies and learning activities, learners employ and engage in different SRL phases, which contribute to learning achievement (Broadbent and Poon, 2015). Context is an integral part of SRL which shapes students’ learning (Winne, 2010). Consequently, students may use new operations as contexts evolve.

Using think-aloud data is a valid approach to uncover SRL processes (Veenman, 2013; Greene et al., 2018). The think-aloud method captures students’ utterances of their activities as they occur, thereby generating data that can be modeled to reflect the dynamic nature of SRL processing (Greene et al., 2018). Event-based data, in this case, think-aloud protocols measured during learning, are particularly suited for investigation of SRL processes (Reimann et al., 2014). Applying process analysis gives us the opportunity to investigate learning processes as they unfold (Molenaar and Järvelä, 2014). Frequency analysis through statistical methods does not allow us to identify how SRL activities are used during learning and how the activities are arranged with respect to their temporal structures (Reimann, 2009). In this respect, educational process mining on the basis of think-aloud data enables a more fine-grained analysis and a deeper understanding of SRL processes (Sonnenberg and Bannert, 2015; Engelmann and Bannert, 2019). Although students’ SRL is highly contextualized (Winne, 2018), there are consistent findings of the link between SRL activities, specifically metacognitive activities, and learning outcomes (Bannert and Mengelkamp, 2013; Bannert et al., 2014; Sonnenberg and Bannert, 2015; Müller and Seufert, 2018) suggesting that some processes are consistently beneficial to learning across different learning tasks and contexts, such as monitoring and better integration of task analysis processes including orientation, planning, and goal specification. In order to model SRL processes meaningfully, researchers in previous studies have selected representative groups of students, such as successful and less successful students (e.g., Schoor and Bannert, 2012; Bannert et al., 2014; Engelmann and Bannert, 2019; Huang and Lajoie, 2021). However, differing approaches and the corresponding analyses among prior studies investigating the unfolding of SRL processes during learning without standardized guidelines (e.g., types of data used, how learning events have been coded and their granularity, modeling methods) pose a challenge for generalizing findings. In the study reported in this paper, we investigated SRL processes which took place in an online learning environment by comparing SRL activities of successful and less successful students. Furthermore, we extended contributions from past research also conducted in digital and online learning settings by looking at the replicability of findings of SRL activities across learning contexts and tasks through the utilization of a similar approach as a previous study to ascertain if some SRL processes are consistently beneficial for learning. The general aim of the paper was to identify strengths and deficits of student’s SRL activities by collecting and analyzing think-aloud data in order to build the basis for future SRL interventions.

### Models and Components of SRL

Learners regulate their learning by monitoring and controlling the processing of content and operations they apply to content’s processing as they pursue goals to augment and edit prior knowledge (Winne, 2019). SRL is a process, whereby learners employ various cognitive strategies in an effective manner, which is directed by their metacognitive knowledge and skills (Boekaerts, 1999). Although there are several SRL models available (e.g., Winne and Hadwin, 1998; Pintrich, 2000; Zimmerman, 2000) offering different perspectives, they share the common assumption of SRL defined as cyclical phases comprised of several processes (Puustinen and Pulkkinen, 2001; Panadero, 2017). Based on comparison of different SRL models of Puustinen and Pulkkinen (2001), there are three common identifiable phases in the SRL process, namely the preparatory, performance, and appraisal phases. In the preparatory phase, learners analyze the task, set goals, and strategically plan their learning. Goals are the set of standards students refer to in order to monitor their learning metacognitively during the learning process. They also guide students in forming their plan (Winne, 2018). Strategic planning refers to activating prior knowledge, and analyzing the task in order to determine which cognitive strategies to use (Pintrich, 1999). During the performance phase, students monitor and regulate their learning as they employ cognitive strategies to perform the task, which are guided by their goals. Regulating of learning *via* monitoring and control processes plays a paramount role which is further elaborated by Nelson and Narens (1994). According to them, cognition is structured into a “meta-level” and an “object-level.” A mental representation of one’s cognition forms the meta-level; one’s cognition is therefore the object-level. Monitoring and control are regulatory processes that reflect the interaction between the meta- and object levels. Monitoring leads to the mental representation of one’s cognition and control processes alter the object-level (i.e., one’s cognition). Dependent on the meta-level representation (e.g., judgments of their learning) derived from monitoring, one could modify control processes, such as through rereading the text or terminate the current strategy. Weinstein and Mayer (1983) identified three main cognitive strategies – rehearsal, elaboration, and organization – which are important for academic performance. Rehearsal strategies, which reflect shallow processing, enable students to take note of important information, which are kept in their working memory. For example, students may repeat out loud what they have read, or take verbatim notes. Elaboration and organization strategies involve students processing information at a deeper level. Elaboration strategies include summarizing, paraphrasing, explaining, creating analogies, etc. Organization strategies include outlining and organizing material learned, such as mapping out and connecting ideas. In the final SRL phase, known as appraisal, students evaluate and reflect on their learning which in turn lead to adaptations for their next learning cycle. Evaluation refers to one’s comparison of current progress with a pre-defined goal or standard (Zimmerman, 2000). In a more elaborated model such as the COPES model (Winne and Hadwin, 1998), monitoring is also assumed to be omnipresent across all phases, which leads to control processes that reduce discrepancies between current progress and standards.

Most SRL models explain regulatory processes to occur in a time-ordered sequence but not in a specific stringent order (Azevedo, 2009). In past empirical studies investigating the temporal structure of students’ SRL activities (Bannert et al., 2014; Sonnenberg and Bannert, 2015; Paans et al., 2019; Cerezo et al., 2020; Huang and Lajoie, 2021), students’ SRL processes in the main SRL phases were distinguishable, particularly among students who were more successful. Moos and Miller (2015) also found that the SRL processes, planning and monitoring, are more stable across learning tasks. Therefore, in the current study, we investigated the consistency of findings of students’ SRL activities and by distinguishing successful and less successful students.

### SRL and Learning Performance

Self-regulated learning has been shown to be related to academic performance, especially transfer test scores (Schunk and Greene, 2017). Students transfer their knowledge when they apply knowledge and skills to a new situation or problem (Bloom et al., 1956). Metacognitive activities can help to deepen understanding (Bannert et al., 2009). Specifically, metacognitive activities comprise of analyzing the task through orientation, planning, and setting goals for learning, regulation of cognitive activities, monitoring the processing of content and operations applied for the content’s processing, and evaluation of learning (Meijer et al., 2006; Schunk and Greene, 2017). Deekens et al. (2018) found in two studies they conducted that monitoring activities, which are part of metacognitive activities, were positively associated with use of deep learning strategies. The association between metacognitive activities and learning has been repeatedly found by empirical studies investigating (and supporting) SRL activities and learning performance, with effects found particularly in transfer performance. Bannert and Mengelkamp (2013) conducted three experimental studies using a range of metacognitive prompts to support university students’ SRL when learning with hypermedia. They investigated students’ metacognitive activities and analyzed SRL processes. They found that experimental groups which were supported by metacognitive prompts engaged in more metacognitive activities. Furthermore, they found significant effects of metacognitive prompts on only transfer performance in two out of three studies. Bannert et al. (2014) similarly found significant positive correlation between metacognitive activities and transfer performance in a study, which measured students’ learning activities with the use of think-aloud protocols. Sonnenberg and Bannert (2015) investigated the learning activities which contributed to differences in transfer performance between an experimental group supported by self-directed prompts and a control group. Their findings indicated that transfer performance was mediated by the number of metacognitive events. In particular, monitoring activities seemed to have been the driving force of the mediation (i.e., larger effect of monitoring when compared to effect of all metacognitive events) in the experimental group supported by metacognitive prompts. In the experimental study by Müller and Seufert (2018), they observed the effects of self-regulation prompts for the purpose of activating self-regulation activities on university students’ learning across two learning sessions. Their findings revealed significant differences between the groups in terms of transfer performance after the first session, where students received prompts. These studies highlighted the role of SRL, particularly metacognitive activities, in improving students’ transfer performance.

The success of students’ learning is dependent on the skills in applying strategies in their SRL activities during learning. Yet, students are not able to produce the skills required in a spontaneous manner in a phenomenon termed production deficiency (Flavell et al., 1966). Understanding how the spontaneous unfolding of SRL activities occurs could provide us better insights on how and at which points to support students’ learning. Therefore, there is a need to examine not only learning outcomes, but also the processes during learning.

### Measuring SRL Processes Using the Think-Aloud Approach

Self-regulated learning processes have been measured in several ways through self-report questionnaires (e.g., Motivated Strategies for Learning Questionnaires; Pintrich et al., 1993), think-aloud protocols (Johnson et al., 2011; Bannert et al., 2014; Vandevelde et al., 2015), micro-analyses (Cleary and Callan, 2017; Dörrenbächer-Ulrich et al., 2021; Kia et al., 2021), and increasingly, trace data (Järvelä et al., 2019; Cerezo et al., 2020; Huang and Lajoie, 2021). The reliability of self-report questionnaires in measuring SRL has been repeatedly questioned (Greene and Azevedo, 2010) and they have been shown to be poor predictors of actual SRL behavior (Bannert and Mengelkamp, 2013; Veenman, 2016). Self-report questionnaires used are typically administered offline and measure global use of SRL strategies, which show low calibration with actual SRL behavior, though online micro-analytic (i.e., fine-grained) self-report questionnaires may show better calibration with actual SRL behavior indicators (Rovers et al., 2019). Online methods such as think aloud are strong predictors of achievement (Veenman, 2013). This could be explained by the finer grained nature of these measures (e.g., think-aloud protocols, micro-analytic questions, trace data, and eye-tracking data), in comparison with self-reports which focus on global SRL rather than specific strategies (Rovers et al., 2019). Greene et al. (2010) analyzed SRL activities of university students in a hypermedia learning environment, while they were thinking aloud. They found that measures of SRL which were coded from the think-aloud protocols using a previously established coding scheme were more advantageous than self-report instruments. Heirweg et al. (2019) used two different methods (i.e., think-aloud protocols and self-reports) for the exploration of SRL profiles in primary school students. Their findings supported past research that students overestimate their SRL behavior in self-reports. From a methodological perspective, measuring SRL using think-aloud data has been shown to be more insightful than self-reports as it helps better in determining SRL activities and learning achievements. Although research using trace data, especially with the combination of multimodal and multichannel data (e.g., logs and eye tracking, etc.) has been gaining popularity due to benefits over self-report questionnaires, working with these data comes with specific challenges, as summarized by Azevedo and Gašević (2019), such as temporal alignment of data, variations in data granularity, theoretical assumptions, and interpretations of different data streams, and so forth. Thus, in our study, we focus on the use of think-aloud protocols using an established coding scheme to measure SRL processes.

#### Using Concurrent Think Aloud to Make Learning Activities Observable

Using concurrent think aloud (CTA) is a powerful approach to observe and model the dynamic nature of SRL processes (Greene et al., 2018). CTA allows students to verbalize every thought out loud without additional processing such as interpretation or judgment (Ericsson and Simon, 1984). To maximize the rigor of this approach, pre-requisites of studies implementing CTA are both adequate prior training and prompting during the session (Hu and Gao, 2017; Greene et al., 2018). This means that participants should be allowed to practice in an appropriate manner in order to familiarize with the procedure and that experimenters are required to prompt participants to continue thinking aloud in the event of silences. The disadvantage of using CTA is the additional processing time required by participants, especially with verbal encoding processes (Ericsson and Simon, 1984). Extended silences could indicate the high cognitive load participants are experiencing at the moment or the activity they are performing is highly automated (Ericsson and Simon, 1984; Elling et al., 2012). Another downside of working with think-aloud protocols is that the coding process is a labor-intensive procedure. Despite its limitations, using CTA allows researchers to see the inner workings of how learners process information as it does not alter information processing (Winne, 2018).

It is, however, important to note for whom CTA gathers valid observations and in general, whether it has a reactive effect. In review of Hu and Gao (2017) on past studies on the reactive effects of think aloud as a method, their findings suggested that older students (i.e., university students) were less inclined to alter their processes when asked to think aloud as compared to younger students such as primary school students. In meta-analysis of almost 3,500 participants in 94 independent data sets of Fox et al. (2011), they found that the use of CTA did not lead to performance changes. Bannert and Mengelkamp (2008) found no performance differences between students who were asked to think aloud during learning and the control group who learned in silence. Finally, since the activities students are engaged in are deduced from their verbalizations, the coding process calls for the use of sophisticated coding schemes derived from theory and the procedure to be performed by trained raters (Greene et al., 2018). In conclusion, CTA is a valuable method to measure SRL processes in a nonreactive manner.

### Using Process Mining to Investigate SRL Processes

Analyzing sequences of actions learners take while learning provides an opportunity to investigate SRL processes beyond learning outcomes (Roll and Winne, 2015). This has led to increased use of approaches in identifying SRL patterns by means of process mining, sequence mining, t-pattern analysis, lag sequential analysis, statistical discourse analysis, and so forth (Molenaar and Järvelä, 2014); process mining provides insights into the temporal structures of students’ SRL (Bannert et al., 2014) and hence, its use in the educational context for the purpose of discovery and conformance checking of learning processes is one of the top five uses of process mining (Garcia et al., 2019). The conceptualization of SRL as a series of events which develops and unfolds over time has led to growing research exploring the temporal and sequential sequences of SRL (Molenaar and Järvelä, 2014). The variable-centered approach of frequency analysis of SRL occurrences using statistical methods assumes that independent variables are constantly acting upon the dependent variables (Reimann, 2009). As an addition to the variable-centered approach, the event-based approach in SRL research aims to increase explanatory power by identifying how SRL processes occur and develop over time (Reimann, 2009; Molenaar, 2014).

Think-aloud protocols are one way to measure SRL using the event-based perspective, whereby SRL is observed as a sequence of temporal events (Winne and Perry, 2000). Bannert et al. (2014) analyzed the process patterns of successful and less successful students who learned in a single session in a hypermedia learning environment using their think-aloud protocols as indicators of their SRL behavior. Their findings revealed that successful students not only showed more learning and regulation activities, but there were also differences in temporal structures, which were detected in the process models generated from applying the Fuzzy Miner algorithm on coded think-aloud protocols. They found that successful students engaged in preparatory activities prior to learning, learned more deeply by engaging in deeper cognitive processes such as elaboration, and evaluated their learning. They also continuously monitored various learning activities throughout their learning. In contrast, less successful students adopted a surface approach to learning, whereby superficial cognitive activities such as repetition were more dominant in their process model. Evaluation activities were notably absent from the model.

Other studies focused on using logfiles to detect SRL patterns. Although, the data streams differed, the goal was similar – investigating SRL processes by means of students’ learning activities. Huang and Lajoie (2021) identified SRL patterns in teachers’ acquisition of technological pedagogical content knowledge (TPACK) in a computer-based learning environment (CBLE). They collected log files which included how teachers navigated in the learning environment, as well as lesson plans which were evaluated. On the basis of TPACK application quality from the lesson plans and teachers’ self-report of TPACK, they distinguished three groups which represented low to high achievements. They then applied the Fuzzy Miner algorithm for the detection of SRL patterns within and across groups. On a global level, the groups exhibited differences in how SRL activities took place. In particular, the model of high TPACK achievers showed that all SRL events were connected and they began their learning by analyzing the task and setting goals. They constantly monitored as they were performing the tasks. Additionally, when comparing high and low clusters within each group, high clusters showed iterative SRL patterns and the dominant role of monitoring of various activities.

Other than studies which were conducted in a single session in the lab mentioned above, process mining has also been utilized in other learning contexts, such as online courses. Cerezo et al. (2020) applied the Inductive Miner algorithm to discover SRL patterns in a university e-Learning course that stretched over a semester with over 100 students. They analyzed logfiles obtained from the learning platform and concentrated on the process models for passing and failing students and especially in one learning unit. They found that students who failed in this unit displayed SRL patterns which was not supported by neither SRL skills nor instructor recommendations. Students who passed had a combination of more meaningful activities such as comprehension, learning, execution, and reviewing, which demonstrated more effective SRL. Using a larger data set and additionally self-reports, Maldonado-Mahauad et al. (2018) found differences between students who completed a Massive Open Online Course and those who did not. Students who completed the course showed higher engagement with course assessments, and moreover, those who have a higher SRL profile, interacted more deeply with the materials and were more strategic in their learning.

The studies mentioned indicate similarities in the approaches used to uncovering SRL patterns, such as by comparing process models between successful and less successful groups of students. However, the challenges with comparing findings and identifying students’ gaps in SRL across studies lie in the learning contexts and tasks, types of data used (e.g., think-aloud protocols, logfiles, and self-reports) as well as how learning events have been coded and their granularity. Therefore, we reflect upon our findings mainly with the study from Bannert et al. (2014) owing to the data type (i.e., think aloud) used, and coding scheme which is an adapted version of the coding scheme they used. We adopted a similar approach using the Fuzzy Miner algorithm and the parameters used in their study. Further, Saint et al. (2021) compared four prominent process mining algorithms used in SRL research, namely, Inductive Miner (Leemans et al., 2014), Heuristics Miner (Weijters et al., 2006), Fuzzy Miner (Günther and Van Der Aalst, 2007), and pMiner (Gatta et al., 2017). They systematically explored the insights provided by each of the process mining algorithms in the context of SRL research and found that Fuzzy Miner holds the highest value for interpreting SRL processes with clarity. Therefore, we assume that using Fuzzy Miner on coded think-aloud protocols is an appropriate approach to discover the key SRL processes which take place (or do not take place) during learning.

### The Present Study

Students’ SRL is dependent on the learning task and context (Winne, 2018), but Moos and Miller (2015) have also found that SRL processes, such as planning and monitoring, are more consistently helpful for learning across different tasks. The consistent findings of SRL activities on transfer performance suggest the presence of processes that are beneficial across different learning contexts and tasks. Yet, comparison of findings from previous studies on how SRL processes unfold during learning can be a challenge due to differing approaches and methods. Hence, replication studies are necessary in order to generalize findings. In our study, we sought to find a more stable picture on SRL processes and learning outcomes. The findings of our study extended previous SRL research in digital and online learning settings and provided insights to whether previous findings were replicable, and whether our findings were still valid and coherent to older studies when applied to a different learning task and context. By doing so, we illustrated how we analyzed SRL processes in the online learning environment. Through these findings, we sought to identify students’ gaps in SRL in order to develop better scaffolds by modeling successful and less successful students’ SRL patterns. Building on this, the generated findings were further connected with those of previous process mining studies from the research field. These research questions guided our study:

#### How Do Students (Spontaneously) Regulate Their Learning Activities?

Since there are similarities between our and study of Bannert et al. (2014), and that the learning task required students to engage in SRL activities across all SRL phases, we expected that we observed activities in all categories of SRL. In study of Bannert et al. (2014), students engaged in higher frequencies of metacognitive activities and monitoring activities had the highest frequencies. Other activities such as planning, goal specification, evaluation, and motivation had lower frequencies.

#### How Do Learning Activities and Their Regulation Correspond to Learning Performance?

Based on past studies (Bannert and Mengelkamp, 2013; Bannert et al., 2014; Sonnenberg and Bannert, 2015; Müller and Seufert, 2018), increased metacognitive activities led to better transfer performance. Additionally, better transfer performance was mediated by monitoring activities (Sonnenberg and Bannert, 2015) in the experimental group supported by metacognitive prompts. We anticipated that metacognitive activities had a positive correlation with transfer performance.

#### How Do the Temporal Structures of Learning Activities of Successful and Less Successful Students Differ?

Our study used process mining for the exploration of temporal structures of SRL activities between successful and less successful students. As we aimed to explore the consistency of findings from SRL patterns with past studies, we did not specify any hypothesis related to this research question. However, in general, we expected that similar to Bannert et al. (2014), successful students show SRL patterns closer to those proposed by SRL theories, where the main SRL phases are closely linked.

## Materials and Methods

### Participants

Our study consisted of 36 participants from various universities in Germany. Due to poor quality in the recording of audio data and data loss, we had usable think-aloud data for 32 participants (*M_age_*=26.56years, *SD_age_*=4.18years, 66% female). We then performed a check on the proportion of participants’ think aloud for the whole session and excluded two participants who were thinking aloud for less than 50% of the session to make sure only valid protocols were included. Additionally, we removed one non-native speaker. The final sample consisted of 29 participants. Participation was voluntary – all participants signed a printed consent form – and the participation criteria were that students had German as first language and were studying in a university. The participants were reimbursed with 15 euros for their participation. The participants studied a diverse range of degree majors – 25 in total – such as informatics, education, business administration, chemistry, engineering, law, and political science.

### Design

**Figure 1:**
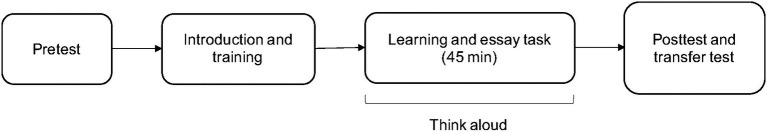
Research design in our present study.

We conducted the study in single onsite sessions with participants individually. Before the session started, participants filled out a demographic questionnaire asking them for their gender, age, and degree major. We used a pre–post-design (see Figure 1), whereby participants completed a domain knowledge test before and after learning. During the 45-min learning phase, where participants had to think aloud, they were tasked to learn and write an essay. Afterward, they completed a transfer test.

#### Learning Environment and Materials

**Figure 2:**
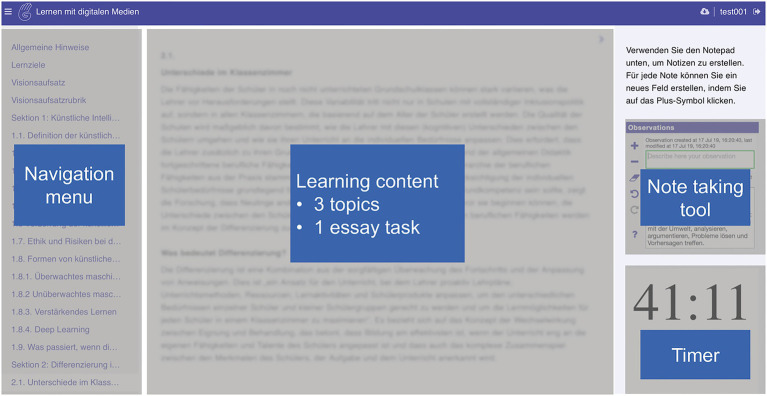
The computer-based learning environment (CBLE).

A CBLE presented learning materials from three topics: Artificial intelligence, differentiation in a classroom, scaffolding, and an essay task. All materials were presented in German. The CBLE (see Figure 2) comprised of a navigation menu, a reading panel, a note-taking tool, and a countdown timer. The CBLE consisted of 37 pages, including one instruction page, an essay-writing page, an essay rubric page, three table of contents pages, one for each topic, and pages presenting learning content. Some irrelevant materials were included in each topic so students had to learn strategically. Participants navigated by clicking the title of the pages on the navigation menu. They could create, edit, and delete notes *via* the note-taking tool. The countdown timer displayed a countdown from 45min.

The learning content contained 5,237 words and six figures. A text readability analysis (Michalke, 2012) showed the Flesch-Kincaid grade-level score and Flesch Reading Ease values (the equivalent for German texts is Amstad) of all three texts to be suitably challenging for university students. The text about artificial intelligence had a Flesch–Kincaid grade-level score of 15.56, and a Flesch Reading Ease value of 41.42. The text about differentiation in the classroom had a Flesch-Kincaid grade-level score of 21.33, and a Flesch Reading Ease value of 22.41. The text about scaffolding had a Flesch-Kincaid grade-level score of 19.84, and a Flesch Reading Ease value of 28.39. The Flesch-Kincaid grade-level score corresponds to grade levels and the higher the number, the more difficult to read is the text. The Flesch Reading Ease value has a range from 0 to 100, with lower numbers indicating more difficult reading.

### Procedure

The data collection session was conducted in a lab at a German university and lasted approximately one and a half hour with an experimenter present throughout. Participants were specifically instructed prior to starting that they would not have sufficient time to read all the materials and write the essay and they would have to learn efficiently and choose what and how they learn. We presented the learning environment on a 23.8-in monitor using a web browser. Audio data were collected *via* a clip-on microphone. Participants had access to a keyboard and mouse at all times.

The session consisted of four parts. Part 1 began with the demographic questionnaire and then pretest questions. All participants were given a maximum of 20min to complete the pretest. In Part 2, the experimenter first introduced the learning environment and tools. Then, the experimenter demonstrated how to think aloud while navigating the learning environment. Finally, the participants were asked to think aloud and complete short exercises, where they navigated through the learning environment and used the tools. At the end of the training, the experimenter would provide a short feedback (e.g., volume needs to be louder). This took between 10 and 15min. In Part 3, the learning phase, participants had 45min to read the text and write an essay. During this phase, they were free in how they navigated or used the tools in the learning environment. However, they had to read and think aloud throughout. Whenever they were silent for more than 5s, or spoke quietly, they were prompted verbally by the experimenter. Finally, in Part 4, participants completed the posttest and transfer test in this sequence. They were given a maximum of 30min for Part 4.

### Instruments

We developed the learning performance measures based on Bloom’s taxonomy of cognitive learning objectives (Bloom et al., 1956). The items in the domain knowledge test were focused on the lower levels of the taxonomy, such as comprehension of the texts, while the transfer test items were focused on the higher levels, such as application of concepts (in the medical field). All items in the tests were compulsory and could not be skipped. The domain knowledge test addressed knowledge relating to sections of the text relevant to the learning goals and consisted of 30 multiple-choice items (ω=0.75). The omega coefficient (ω) has been found to be a better choice than Cronbach’s alpha, especially when scores are normally distributed, as was the case in our study (Trizano-Hermosilla and Alvarado, 2016) and is less prone to over- or underestimation of reliability (Dunn et al., 2014). An example of a domain test item was: “How can an algorithm work better?” with options, (A) “By making the series longer,” (B) “By building in more supervision,” (C) “By analyzing more data” (correct answer), and (D) “By simulating more human behavior.” The sequences of both pre and posttest items were randomized, so that items were not presented in the same order for the pre and posttests, and that items relating to the same topic did not appear together. Each item consisted of four answer options with one correct answer; each correct answer was worth one point with a total of 30 points for the whole test. We measured transfer knowledge with 10 multiple-choice items (ω=0.44). An example of a transfer test item was: “Which of the following describes how artificial intelligence has been used by the healthcare industry?” with options, (A): “Using augmented reality architecture systems to develop quicker and more efficient paths for transporting patients at the emergency department,” (B): “Using natural language processing to analyze thousands of medical papers for better informed treatment plans” (correct answer), (C): “Automatic transfer of patient information whenever another hospital requests for it.,” and (D): “Using robots to prepare meals that meet patients’ treatment and dietary needs as indicated in the patient file.” Identical to the format of the domain knowledge test, there were four options provided with one correct answer. Each correct answer was awarded one point with the total points possible being 10 points. Participants were asked to apply their knowledge of artificial intelligence in the medical field.

#### Essay Task and Coding

To elicit self-regulated learning strategies, participants were tasked to write a 300–400-word essay during the 45-min learning phase of the session. Detailed task instructions and an essay assessment rubric were provided in the learning environment. The task was to apply what they had learnt from all three text topics into an essay, where they envision and suggest how learning in schools would look like in the year 2035. The essays were assessed manually by two trained coders and inter-rater reliability (weighted *κ*=0.68) was calculated by randomly selecting 17 essays. According to Fleiss et al. (2003), a weighted kappa value is interpreted the same way as a kappa value and the value, we have obtained was acceptable to proceed.

### Think-Aloud Procedure and Coding Scheme

All verbalizations in the learning phase were coded using a coding scheme (see Table 1) adapted from prior research (Bannert, 2007; Molenaar et al., 2011; Sonnenberg and Bannert, 2015). In their theoretical framework, the authors characterized hypermedia learning into three main categories, Metacognition, Cognition, and Motivation. Furthermore, there are distinct sub-categories within the larger categories of Metacognition and Cognition. Table 1 presents the coding categories, description, and examples. Metacognition included the subcategories, *orientation*, *planning*, *monitoring*, *search*, and *evaluation*. Cognition was further categorized into *reading*, *rereading*, *superficial processing*, *elaboration*, and *organization*. All verbalizations relating to motivational aspects of the task, situation, or self, were coded as *motivation*. The final category, *other*, included irrelevant utterances, or segments which were incomprehensible (e.g., a mumble).

**Table 1:** Think-aloud coding scheme adapted from Bannert (2007).

Initial coding category	Final coding category	Final code	Examples
Metacognition			
Orientation	Task analysis	ANALYSIS	I have to write an essay
Planning	Task analysis	ANALYSIS	First, I will read the text through.
Goal specification	Task analysis	ANALYSIS	I must first understand Scaffolding.
Monitoring	Monitoring	MONITOR	Okay, I understand it.
Search	Search	SEARCH	I’m looking for the concept, “Divergence.”
Evaluation	Evaluation	EVAL	I think I have completed the learning goals.
Cognition			
Reading	Reading	READ	Reading content out loud
Rereading	Rereading	REREAD	Rereading content out loud
Superficial processing	Repeating	REPEAT	Rehearsal, writing verbatim notes from content
Elaboration	Elaborating	ELABORATE	That means, the students should be taught in smaller groups.
Organization	Organizing	ORGANIZE	Outlining important points as notes
Motivation	Motivation	MOT	I do not like writing essays.
Other	Rest	REST	I have problems with the mouse.

Two trained research assistants coded the verbal protocols together using the procedure suggested by Chi (1997). Due to economic reasons, segmentation and coding were carried out in one step. Segmentation was performed based on meaning and multiple or nested codes were not allowed. In the event of uncertainty, the final code was decided after discussion with the first author. We found interrater reliability of *κ*=0.95, representing excellent agreement (Fleiss et al., 2003).

### Data Analysis

To answer the research questions we posed, we utilized three approaches. In order to find out how students regulated their learning activities, we performed descriptive analyses of coded think-aloud events to investigate whether and which activities were captured. We also checked if students had learned by conducting a paired samples *t*-test with pre and posttest scores. For our second research question, we correlated the frequencies of activities observed *via* coded think-aloud protocols and the performance measures (i.e., comprehension and transfer tests, and essay). We conducted the Spearman’s correlation due to the non-normal distribution of almost all categories of the coded activities. It is typical of this measurement method (i.e., think aloud) of SRL processes to have non-normal distributions (Greene et al., 2018). Since we expected metacognitive activities to be positively correlated with learning performance, one-tailed correlation analysis was performed for metacognitive activities and learning performance, and two-tailed correlation analysis for the rest of the analyses. Furthermore, we checked how the same SRL activities simultaneously predicted the posttest, transfer, and essay performance by means of multivariate regression analyses. For the third research question, we used the process model discovery algorithm, Fuzzy Miner (Günther and Van Der Aalst, 2007), on two groups of students categorized as successful or less successful, we created prior to the analyses. Successful learning was operationalized by using transfer performance and a median split was conducted to form the groups.

#### Process Discovery Using the Fuzzy Miner Algorithm

We imported the event logs of all coded think-aloud protocols into the ProM process mining framework version 5.2 (Verbeek et al., 2011). All event logs contained a participant ID, timestamp which indicated the start of the activity, and the activity which was coded. We then applied the Fuzzy Miner algorithm (Günther and Van Der Aalst, 2007) on the imported event logs to create two process models – one for the successful group and one for the less successful group. According to its developers, this process mining algorithm is suitable for unstructured real-life data (i.e., coded think-aloud data in our case) and produces meaningful process models which can be interpreted. Fuzzy Miner uses two key metrices to compute the process model which contains nodes and edges, and their respective significance and correlation values. *Significance* refers to the relative level of importance of the observed events and the relations between these events (i.e., edges); *Correlation* refers to “how closely related two events following one another are” (Günther and Van Der Aalst, 2007, p. 333). There are three ways guiding the process simplification approach. The process model retains highly significant events; less significant but highly correlated events are aggregated into clusters. Finally, events which are both less significant and lowly correlated are removed from the model. For our analyses, we used the following parameters as per the modeling procedure by Bannert et al. (2014): edge filter cut-off set at 0.2, utility ratio set at 0.75, node filter set at 0.75 and the significance cut-off set at 0.25. For the purpose of investigating the temporal structure of SRL activities in our model, we excluded the categories, motivation, and others.

## Results

### Frequency Analysis of all Coded SRL Events

In order to answer RQ1, that is, how do students spontaneously regulate their learning activities, we calculated the descriptive results for all coded learning events (see Table 2). We coded a total of 17,477 activities in the 45min learning session for the sample. On average, there were 244 metacognitive and 310 cognitive activities. The participants showed a mean of less than two motivation activities and 45 other utterances, which were not related to learning. Monitoring activities (*M*=159.48, *SD*=65.03) had the highest mean frequency across all categories, followed by reading (*M*=93.21, *SD*=42.69), and elaboration (*M*=90.45, *SD*=69.16). Evaluation activities (*M*=0.62, *SD*=1.63) had the lowest frequency, as well as motivation (*M*=1.83, *SD*=2.61) and Search (*M*=3.52, *SD*=3.63).

**Table 2:** Descriptive table of coded think-aloud events, *n*=29.

	Min	Max	*M*	*SD*
Metacognition				
Orientation	23	147	67.24	31.41
Planning	0	33	10.28	9.22
Goal specification	0	27	3.55	6.03
Monitoring	61	313	159.48	65.03
Search	0	11	3.52	3.63
Evaluation	0	6	0.62	1.63
*Sum of metacognitive activities*	141	373	244.69	73.54
Cognition				
Reading	30	207	93.21	42.69
Rereading	1	156	55.28	37.74
Superficial processing	0	56	4.38	11.66
Elaboration	6	299	90.45	69.16
Organization	0	244	67.59	57.46
*Sum of cognitive activities*	122	477	310.9	81.02
Motivation	0	9	1.83	2.61
Others	8	123	45.24	23.21
*Sum of all coded activities*	313	870	602.66	125.96

### Correlation Analysis of all Coded SRL Events and Learning Outcomes

For RQ2, that is, how do learning activities and their regulation correspond to learning performance, we analyzed the correlations between coded think-aloud events and performance scores. An analysis of pre- and post-knowledge tests using a paired samples *t*-test showed a significant learning gain. We found a significant difference in the pre-knowledge scores (*M*=14.14, *SD*=3.35) and post-knowledge scores (*M*=17.14, *SD*=3.72); *t*(28)=5.87, *p*<0.001, *d*=1.09. The effect size was large (Cohen, 1992). Table 3 presents the descriptive statistics for all learning outcomes.

**Table 3:** Descriptive table of learning outcomes, *n*=29.

	Min	Max	*M*	*SD*
Pretest^a^	7	20	14.14	3.35
Posttest^b^	8	25	17.14	3.72
Transfer^c^	2	10	5.97	1.57
Essay^d^	0	17	8.45	4.31

Maximum possible scores for each learning measure are indicated below.

a, b 30 points.

c 10 points.

d 21 points.

Table 4 presents the correlation results for SRL activities and different learning performance scores. As expected, metacognitive events had no significant correlation to all learning measures except transfer score (*r_s_*=0.37, *p*=0.024). Of all metacognitive activities, transfer performance was significantly correlated to monitoring (*r_s_*=0.44, *p*=0.008), as we expected, and in addition, to search (*r_s_*=0.40, *p*=0.017) and rereading (*r_s_*=0.48, *p*=0.008). The deeper cognitive activities, elaboration and organization, were found to be negatively correlated (*r_s_*=−0.62, *p*<0.001) to each other. Additionally, as a check for consistency, we conducted a multivariate regression analyses for the same SRL activities and learning outcomes. The results showed search to be a predictor for post-knowledge performance (b*=0.68, *p*=0.029), monitoring to be a predictor for transfer performance (b*=0.47, *p*=0.046), as well as evaluation (b*=0.49, *p*=0.034). Elaboration was a predictor for essay performance (b*=0.63, *p*=0.035).

**Table 4:** Correlations for self-regulated learning (SRL) activities and learning performance, *n*=29.

S. No	Variable	1	2	3	4	5	6	7	8	9	10	11	12	13	14	15	16	17	18
1.	Orientation	—																	
2.	Planning	−0.12	—																
3.	Goal specification	−0.03	0.35	—															
4.	Monitoring	−0.08	0.15	0.24	—														
5.	Search	0.27	−0.01	0.21	0.33	—													
6.	Evaluation	0.07	−0.11	0.23	−0.03	0.18	—												
7.	All metacognitive activities	0.30	0.25	0.36	0.88^***^	0.43^*^	0.01	—											
8.	Reading	−0.06	−0.23	−0.17	0.29	−0.23	0.20	0.25	—										
9.	Rereading	0.24	0.08	0.14	0.03	0.67^***^	0.05	0.17	−0.26	—									
10.	Superficial processing	−0.16	0.17	0.19	0.18	0.16	0.11	0.26	−0.06	0.16	—								
11.	Elaboration	−0.05	0.20	0.20	−0.20	0.01	0.18	−0.19	−0.22	0.38^*^	−0.20	—							
12.	Organization	−0.03	−0.21	−0.14	0.18	0.30	−0.22	0.15	0.20	0.03	0.18	−0.62^***^	—						
13.	All cognitive activities	−0.13	−0.04	0.12	0.24	0.26	0.03	0.22	0.36	0.39^*^	−0.01	0.42^*^	0.23	—					
14.	Motivation	0.47^**^	−0.05	0.07	0.04	0.20	−0.06	0.23	−0.04	0.43^*^	−0.06	0.14	−0.03	0.21	—				
15.	Pre-test	0.04	−0.01	0.05	−0.12	0.19	0.24	−0.15	0.06	0.28	−0.31	0.57^**^	−0.18	0.22	0.15	—			
16.	Post-test	0.24	−0.17	−0.02	0.00	0.57^†††^	0.25	−0.01	−0.03	0.34	−0.38^*^	0.30	0.03	0.19	0.24	0.69^***^	—		
17.	Transfer	−0.20	0.04	0.09	0.44^††^	0.40^†^	0.15	0.37^†^	0.08	0.48^**^	0.27	0.14	0.07	0.18	0.17	0.38^*^	0.25	—	
18.	Essay	−0.06	0.33^†^	0.39^†^	−0.14	0.25	0.28	−0.11	−0.54^**^	0.39^*^	−0.02	0.69^***^	−0.43^*^	0.17	0.10	0.36	0.30	0.21	—

As we anticipated that increased metacognitive activities lead to better learning performance, we used a one-tailed correlation analysis for all metacognitive activities and learning measures and a two-tailed analysis for the rest.

* *p*<0.05, two-tailed.

** *p*<0.01, two-tailed.

*** *p*<0.001, two-tailed.

† *p*<0.05, one-tailed.

†† *p*<0.01, one-tailed.

††† *p*<0.001, one tailed.

### Temporal Structures of Learning Activities of Successful and Less Successful Students

We examined the differences in temporal structures of learning activities between successful and less successful students (RQ3) by using process mining. The details of the preparation and process mining procedures are elaborated in the sections below.

#### Successful and Less Successful Students

Similar to the findings from Bannert et al. (2014), we found correlation coefficients to be the highest between the sum of counts of metacognitive activities and transfer scores, and moreover, the only significant correlation among performance measures. Hence, based on our findings, and also past research findings, we proceeded to use transfer scores to operationalize successful learning. We operationalized successful and less successful groups in terms of transfer performance by first calculating the transfer score median (six points). We then split the sample by assigning students with transfer scores above six points to the successful group and students with transfer scores below six points to the less successful group. This resulted in 10 students in the successful group and nine students in the less successful group. Both groups had similar proportion of utterances in the learning session – average of 72% for both groups.

#### Comparison of Different Learning Outcomes Between Successful and Less Successful Groups

Table 5 shows the frequency of learning performance for the successful and less successful students. Out of all performance measures, only the transfer performance showed a statistical significant difference between the successful group (*M*=7.50, *SD*=0.97) and less successful group (*M*=4.22, *SD*=1.09), *t*(17)=6.92, *p*<0.001. The effect size (*d*=3.18) was largest for transfer performance.

**Table 5:** Comparison of learning measures between successful (*n*=10), and less successful students (*n*=9).

	Successful students (*n*=10)		Less successful students (*n*=9)		*t*	*p*	*d*
	*M*	*SD*	*M*	*SD*			
Pretest^a^	15.70	3.50	12.89	3.98	1.64	0.120	0.75
Posttest^b^	18.80	2.86	16.00	3.94	1.79	0.092	0.82
Transfer^c^	7.50	0.97	4.22	1.09	6.92	<0.001	3.18
Essay^d^	8.30	4.60	6.56	4.13	0.87	0.398	0.40

a, b 30 points.

c 10 points.

d 21 points.

#### Comparing Overview of Coded Think-Aloud Events Between Groups

A preliminary check on both groups showed that the groups were similar on mean proportion of think aloud (successful group: *M*=0.72, *SD*=0.10; less successful group: *M*=0.72, *SD*=0.11). Table 6 shows that the groups differed significantly on the sum of metacognitive activities, and specifically monitoring. Additionally, the groups differed on frequencies of rereading activities. Moreover, in other SRL activity categories coded, successful students had higher frequencies in planning (*M*=11.20), search (*M*=5.20), evaluation (*M*=0.90), elaboration (*M*=81.40), and organization (*M*=82.10). Both groups had the lowest frequency in evaluation activities and highest frequency in monitoring activities.

**Table 6:** Absolute, relative frequencies, and test statistics of coded learning events for successful and less successful students.

	Successful (*n*=10)				Less successful (*n*=9)				*t/U*	*p*	*d*
	*M*	*SD*	Absolute frequency	Relative frequency	*M*	*SD*	Absolute frequency	Relative frequency			
Metacognition											
Orientation	67.60	41.17	676	10.15	73.78	12.64	664	13.60	33^a^	0.356	0.46
Planning	11.20	10.22	112	1.68	10.44	10.33	94	1.92	0.16	0.875	0.07
Goal specification	2.40	3.60	24	0.36	4.44	8.89	40	0.82	−0.67	0.512	−0.31
Monitoring	199.10	62.88	1991	29.9	123.78	58.19	1,114	22.81	2.7	**0.015**	1.24
Search	5.20	3.99	52	0.78	2.11	3.41	19	0.39	1.8	0.089	0.83
Evaluation	0.90	2.02	9	0.14	0.33	0.71	3	0.06	0.8	0.438	0.37
*Sum of metacognitive activities*	286.40	71.30	2,864	43.01	214.9	61.28	1934	39.60	2.33	**0.032**	1.07
Cognition											
Reading	96	20.34	960	14.42	106.9	58.39	962	19.70	43^b^	0.905	0.08
Rereading	72.60	36.64	726	10.9	32.89	28.61	296	6.06	2.61	**0.018**	1.20
Superficial processing	3.20	4.73	32	0.48	6.33	18.63	57	1.17	−0.52	0.613	−0.24
Elaboration	81.40	51.16	814	12.22	70.56	68.25	635	13	0.4	0.698	0.18
Organization	82.10	72.11	821	12.33	68.78	54.33	619	12.67	0.45	0.658	0.21
*Sum of cognitive activities*	335.30	76.73	3,353	50.35	285.4	84.64	2,569	52.60	1.35	0.196	0.62
Motivation	2.20	3.22	22	0.33	1.33	1.80	12	0.25	0.71	0.490	0.33

*p* values in bold are significant.

a, b assumption of homogeneity of variance violated, nonparametric test Mann-Whitney U reported.

#### Process Analysis of Successful and Less Successful Students

We adopted the aggregation approach of Bannert et al. (2014) to reduce complexity and prevent “Spaghetti” process models when applying the Fuzzy Miner algorithm. “Spaghetti” models are overly complex models which pose great challenges for interpretation (Günther and Van Der Aalst, 2007). Similar to their study, we aggregated three metacognitive categories (orientation, planning, and goal specification) into ANALYSIS. They further aggregated the deeper cognitive activities, elaboration, and organization. Additionally, they had only the categories, read, and repeat. However, we opted to retain all cognitive categories due to two main reasons. First, there was a statistically significant negative correlation between elaboration and organization, possibly indicating that they did not belong to one major category of deep processing. In order to find out the temporal arrangements of these activities to unravel why there was a significant negative correlation between these categories of activities, it was necessary to keep them in their individual categories, and not group them together. Second, in our study, instead of only repeat, we differentiated between superficial processing and rereading in order to capture activities related to rereading of the learning materials, notes, and essay, and copying of learning materials by writing verbatim notes. Superficial processing was represented in the model as REPEAT and rereading as REREAD.

**Figure 3:**
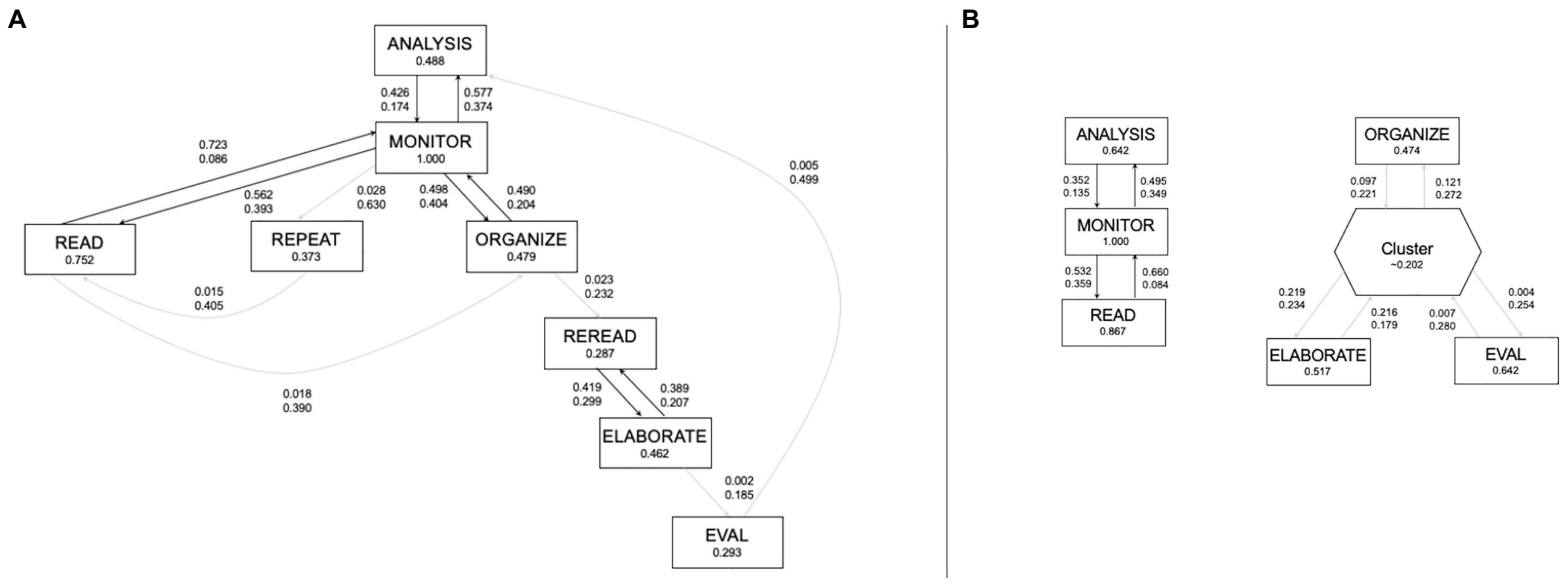
Process models of **(A)** successful (*n*=10) and **(B)** less successful (*n*=9) students. Metacognitive activities are: orientation, planning, and goal specification (ANALYSIS), monitoring (MONITOR), and evaluation (EVAL). Cognitive activities are: reading (READ), rereading (REREAD), superficial processing (REPEAT), elaboration (ELABORATE), and organization (ORGANIZE). The cluster in the right model aggregated REREAD and REPEAT due to low significance values. Significance refers to relative importance of observed events (i.e., nodes) and the relations between them (i.e., edges). Correlation refers to how closely related two consecutive events are. Both values have a maximum of one.

Figure 3 illustrates the process models for successful students and less successful students. In both groups’ model, search was omitted as it did not hit the minimum significance cut-off value of 0.25, as in Bannert et al. (2014). For the successful students’ model, all activities were connected – that is, every activity was connected to at least one other activity. Successful students started with preparatory activities, then they monitored as they performed various cognitive activities (ANALYSIS → MONITOR → READ/REPEAT/ORGANIZE). The model shows a double loop with ANALYSIS, MONITOR, and READ, and a double loop with ANALYSIS, MONITOR, and ORGANIZE. The successful students engaged in monitoring and control activities (MONITOR → READ, MONITOR → ORGANIZE, MONITOR → REPEAT). There was also a chain of cognitive activities students performed, shown as ORGANIZE → REREAD → ELABORATE, whereby REREAD and ELABORATE shared a mutual link. Finally, students evaluated their learning which is then connected back to analysis (EVAL → ANALYSIS). Overall, MONITOR was a dominant SRL activity, whereby it had both a high significance value of one, indicating high frequency and was connected to multiple activities. The model of the less successful students was divided into two groups of activities, with one group containing a cluster of two cognitive activities. A cluster appears in a process model when significance cut-off values are not met and events are aggregated together (Günther and Van Der Aalst, 2007). The model shows that students in this group engaged in preparatory activities as they monitored and read the learning texts (ANALYSIS → MONITOR → READ). Like in the successful group, there were double loops between these activities. The second group of activities showed the deeper cognitive activities, ELABORATE and ORGANIZE, and the metacognitive activity, evaluation (EVAL), weakly linked to the cluster which contained REREAD and REPEAT. Similar to the successful group, MONITOR had a high significance value of one, but in contrast, was linked to only two other activities.

To sum up, monitoring had a high frequency in both groups but the models looked distinctly different; the successful students’ model contained a SRL cycle with all activities connected but the less successful students’ model was disjointed and had deeper cognitive activities and evaluation isolated from analysis and monitoring.

## Discussion

In the study presented, we investigated how students spontaneously regulated their learning, how their learning activities corresponded with learning performance, and the temporal order of SRL activities by means of process mining of *post hoc* coded think-aloud events. We compared our findings to previous studies to search for generalizability through replicability of findings with regard to SRL activities in order to identify gaps for the development of future SRL interventions. Although most of our results were parallel to previous studies, which we expected, they also pointed to some differences. We discuss our findings in more detail with respect to the specific research questions we introduced at the beginning.

With regard to our first research question, we observed activities in all categories of our coding scheme which indicated that students engaged in a range of activities in all three major phases of SRL throughout the learning session. In contrast to Bannert et al. (2014), there were more cognitive than metacognitive activities observed in our study. Frequency of monitoring activities was highest, as in their study. Frequencies of specific activities, such as planning, goal specification, evaluation, and motivation were similarly lower in our study. During the learning session, the students’ main learning task was to read the text and write an essay. This is contrary to the study of Bannert et al. (2014) which had no writing task during learning. Writing is a complex and challenging task which encompasses a variety of recursive cognitive processes (Kellogg, 1987). This could have led to the students engaging in higher frequency of cognitive activities to facilitate the essay writing process, as seen in our study.

In our second research question, we investigated the relationship between SRL activities and learning outcomes by means of correlation analysis. In our analyses, we zeroed in on the transfer performance to establish coherence of findings with past studies (i.e., Bannert and Mengelkamp, 2013; Bannert et al., 2014; Sonnenberg and Bannert, 2015; Müller and Seufert, 2018). Our findings replicated the link between metacognitive activities and transfer performance as we had expected. Additionally, we found the metacognitive activity, search, and the more superficial cognitive activity, rereading, corresponding positively with transfer performance. Transfer performance was subsequently used to distinguish successful and less successful students for the last research question. A secondary finding which we did not expect was the significant negative correlation between elaboration and organization activities. A possible explanation was that due to limited time to complete the task, some students may have opted to prioritize one activity over the other. For example, some students organized their learning by taking notes extensively, while other students focused on elaborating on their learning and ideas in the essay and while learning. However, in order to understand how these activities took place during the learning session, we modeled the temporal arrangement of learning activities for the third research question.

We examined the frequencies and temporal structures of learning activities between successful and less successful students for the third research question. We first compared the frequencies of the two groups and found the SRL activity with the highest frequency for both groups to be monitoring, like in the study of Bannert et al. (2014). We also found similarly low frequencies of evaluation, goal specification, and planning, for both groups of students in our study. Although, our study used different learning tasks and materials, we found comparable patterns in the frequencies of SRL activities. Successful students engaged in higher frequencies of metacognitive activities in conjunction with deeper cognitive activities, resembling the findings by Bannert et al. (2014) who found that successful students adopted a deep level approach to learning. Rereading was also a specific activity that successful students in our study engaged significantly more frequently in than the less successful students, which we did not anticipate as it is typically known to be less effective than other strategies such as elaboration (Dunlosky, 2013). Rereading is a superficial cognitive activity (Weinstein and Mayer, 1983) usually executed by less successful students with lower deep knowledge performance (i.e., transfer; Bannert et al., 2014). Nevertheless, students who perform well include rereading strategically (i.e., when to use it) in the repertoire of activities they perform when learning (Matcha et al., 2019). Moreover, students who are skilled in text reading “not only look for important information, they process that important information differentially (e.g., rereading it, underlining it, paraphrasing it”; Pressley, 2002, p. 295). Selective rereading is often performed by skilled readers (Pressley, 2002). As shown in the process model of the successful students in our study, rereading was preceded by organization, which shared a mutual relation to monitoring. Furthermore, monitoring shared a mutual relation to reading. Successful students read the text, monitored their learning and comprehension, and processed the content deeply *via* organizational strategies and selectively reread parts of the text. Less successful students, on the other hand, read the text, monitored their learning and comprehension, but carried out other cognitive strategies subsequently, such as organization and elaboration, in an unconnected manner. To summarize, our findings indicated that successful students employed rereading strategies differently, in addition to using organization strategies an intermediate learning activity, and better integrated these strategies into their learning.

Although both groups of students utilized organizational strategies with similar frequencies during their learning, their process models revealed differences in the temporal arrangement of their activities. According to Weinstein and Mayer (1986), organizational strategies differ between basic and complex learning tasks. In basic learning tasks, students may group items into categories to remember them better. On the other hand, for complex learning tasks like in our study, students select and connect key ideas as they are learning. For example, in our learning task, successful students took notes and organized what they have learnt while monitoring their learning. Furthermore, good readers monitor their comprehension more than poor readers (Weinstein and Mayer, 1986). In the process model of the successful students, we observed that all activities, cognitive and metacognitive, were linked, as well as both superficial and deep cognitive activities, suggesting that they were able to combine and deploy different strategies during learning. Based on their monitoring, they employed various corrective strategies (i.e., reading, repeating, organizing, rereading, and elaboration). In contrast, despite the less successful students engaging in similarly diverse activities, also deeper processing of information, they demonstrated difficulty in combining these activities, leading to groups of activities which were detached from each other. Particularly, monitoring was not linked to both the deeper cognitive activities and evaluation was not linked to analysis. However, we also observed that more can be done to support the successful students. For example, the link between elaboration and evaluation was weaker and less significant than other processes such as monitor and read, and in comparison with findings from Bannert et al. (2014). To sum up, the process model of the successful students showed more congruence to SRL models proposed from theory, comprising of the three main SRL phases we introduced in the beginning. Based on the differences identified between the process models of the more and less successful students, we are able to identify SRL gaps as a basis for interventions to support SRL.

### Implications for Research and Practice

The findings we have presented illustrated some SRL processes are consistently beneficial for learning across tasks and contexts, while adopting similar methods used in Bannert et al. (2014). By means of doing so, we mitigated issues arising from granularity and categories used in the coding scheme, as well as, parameters applied during process mining. Our findings provide us insights as to which SRL processes are still lacking during learning. For example, less successful students monitor to a high extent based on frequencies, but limited to specific processes (i.e., reading and analysis) as illustrated by their process model. According to Winne and Hadwin (1998), monitoring is fundamental to SRL and occurs throughout all phases, as reflected in the process model of successful students in our study. In the current SRL models (e.g., Winne and Hadwin, 1998; Zimmerman, 2000; Panadero, 2017), students are assumed to engage in activities and processes across different SRL phases in a recursive manner. This was exemplified in the process model of the successful students, and conversely, students who did not do so, were less successful in their learning. We found that SRL needs to be performed in strategic combinations for higher effectiveness with regard to transfer performance. Therefore, our findings corroborated with previous studies which found that students who performed better regulated their learning strategically through a meaningful combination of SRL activities (Saint et al., 2018; Matcha et al., 2019). Our findings set the groundwork for developing scaffolds through the SRL gaps we have identified, which consisted not only of individual activities, but also SRL processes and patterns.

Through our study, we were able to further validate the use of think aloud as an SRL measurement approach through replication of findings from Bannert et al. (2014). Increasingly, researchers in the field of SRL have advocated for the use of nonobtrusive measurement methods and the use of trace data using various combinations of data streams has shown promising advances to detect SRL processes (Winne and Perry, 2000; Siadaty et al., 2016; Taub et al., 2017; Azevedo and Gašević, 2019). However, the issue of validity and reliability of trace data remain a challenge (Winne, 2020). Using current valid measures as presented in our study as the basis for validating other measurement protocols, such as with trace data, is one way to circumvent the issues, as per the procedure from (Fan, van der Graaf, et al., submitted) who used the think-aloud protocols as the “ground truth” for mapping SRL activities measured through trace data.

### Limitations and Future Research

Our present study had a relatively small sample size, particularly in the successful and less successful group. Despite this limitation, we considered our findings meaningful for the following reasons. First, we had a reasonably large number of data points for the coded SRL activities. Second, our results supported previous research findings, and third, we addressed the research aim of identifying students’ gaps in SRL in order to develop better scaffolds. Nevertheless, replication studies should be conducted. Our study investigated the frequency and temporal structure of SRL activities by coding and analyzing presence of these activities. However, we observed that monitoring had a significant role in students’ SRL for both successful and less successful students, but the resulting control measures differed. Our findings indicated that monitoring was linked to four activities in the successful group but limited to two activities in the less successful group. This suggests that the connection of monitoring activities with other activities (i.e., what students monitor) led to differential transfer performance. In tasks which require text comprehension, monitoring is a core skill but metacomprehension (accuracy) tends to be underdeveloped (Prinz et al., 2020) and poor monitoring accuracy have consequential effects on the use of effective control and remediation measures (Serra and Metcalfe, 2009). The present study’s learning task involved intensive text reading and comprehension through which students had to make decisions on what to read, how to read, how to proceed with the materials they have read. However, the quality of monitoring (i.e., how students monitor) was not assessed and could have led to differences in the control measures successful and less successful students carried out. We recommend that future research include further distinction of the quality of monitoring.

Although it has been established that CTA protocols do not interfere with information processing, students are only able to verbalize thoughts that are conscious (i.e., in working memory; Ericsson and Simon, 1984). Wirth et al. (2020) highlighted the issues with assuming that students are consciously self-regulating their learning at all times. Wirth et al. (2020) argued that SRL models should not be limited to active and conscious SRL but also reactive and unconscious SRL which does not induce additional cognitive load. They proposed a three-layer model (i.e., content, learning strategy, and metacognitive) respectively corresponding to the structural components of memory (i.e., sensory, working, and long-term). They propositioned that consciousness of learning and regulation occurs when they reach the working memory, through sustained and strong resonance. The term, resonance, refers to the automatic processing of information in the sensory memory through information-expectations alignment *via* interaction with the long-term memory. Whenever sensory information in a resonant state remains in the sensory memory, interaction can occur with the long-term memory without entering the working memory, thus, contributing to learning and application of SRL strategies without consciousness (and inducing cognitive load on working memory). At the metacognition layer, metacognitive regulation can occur (through resonance) without reaching consciousness. Therefore, using CTA for the measurement of SRL processes poses a challenge for the observation of unconscious SRL. Future studies need to include measurement of SRL activities beyond one methodological approach to fill the gaps present in only one data stream, such as the limitations of CTA. However, it is equally pertinent that SRL activities are measured in a valid manner, hence the complementary roles of different data sources (Fan, Lim, et al., submitted) combined and compared trace data and think aloud and calculated “match rates” – whether SRL activities detected in trace data and think aloud were congruent. They analyzed which SRL activities were observed in both data channels, and also which activities were not observed in one data channel, but the other. Through studies like these, we are able to compensate for the shortfalls of a single measurement approach.

## Conclusion

In conclusion, our present study investigated the consistency of findings when applying new learning contexts and tasks, in terms of SRL processes and learning outcomes, in order to continually improve our understanding of students’ SRL gaps to better design scaffolds. Process models give deeper insight to how SRL activities are connected and arranged, beyond frequency counts. Our findings demonstrated that some processes, especially when occurring in a specific sequence, are consistently beneficial even when learning in a new context and task. Future interventions need to focus not only on repertoire of SRL strategies but also knowing when to use them.

## Data Availability Statement

The raw data supporting the conclusions of this article will be made available by the authors, without undue reservation.

## Ethics Statement

Ethical review and approval was not required for the study on human participants in accordance with the local legislation and institutional requirements. The patients/participants provided their written informed consent to participate in this study.

## Author Contributions

LL performed the conceptualization and design of the study, data collection, coding, and analysis, and writing of the manuscript and revisions. LL and JG designed the learning environment and instruments. MB and IM provided the original think aloud coding scheme and supported the adaption of the final version. MB supervised the conceptualization and design of the study and reviewed the writing and editing. YF, JG, and JK contributed to the revision of the manuscript. MB, IM, DG, and JM conceptualized and designed the project which this study is a part of, and the revision of the manuscript. All authors contributed to the article and approved the submitted version.

## Funding

This work is a result of the FLoRA research project funded by DFG (Germany), NWO (Netherlands), and ESRC (United Kingdom) as part of Open Research Area (ORA) BA20144/10-1, NWO464.18.104, and ES/S015701/1.

## Conflict of Interest

The authors declare that the research was conducted in the absence of any commercial or financial relationships that could be construed as a potential conflict of interest.

## Publisher’s Note

All claims expressed in this article are solely those of the authors and do not necessarily represent those of their affiliated organizations, or those of the publisher, the editors and the reviewers. Any product that may be evaluated in this article, or claim that may be made by its manufacturer, is not guaranteed or endorsed by the publisher.
